# Layer 6 cortical neurons require Reelin-Dab1 signaling for cellular orientation, Golgi deployment, and directed neurite growth into the marginal zone

**DOI:** 10.1186/1749-8104-7-25

**Published:** 2012-07-07

**Authors:** Ryan S O’Dell, Candida J M Ustine, David A Cameron, Sean M Lawless, Rebecca M Williams, Warren R Zipfel, Eric C Olson

**Affiliations:** 1 Department of Neuroscience and Physiology, SUNY Upstate Medical University, 3295 Weiskotten Hall 750 E. Adams St, Syracuse, NY, 13210, USA; 2 Department of Biomedical Engineering, Cornell University, Ithaca, NY, 14853, USA; 3 MD/PhD Program, SUNY Upstate Medical University, Syracuse, NY, 13210, USA

**Keywords:** Preplate, Neurite, Orientation, Epilepsy, Migration, Cortex, Dendrite

## Abstract

**Background:**

The secreted ligand Reelin is believed to regulate the translocation of prospective layer 6 (L6) neocortical neurons into the preplate, a loose layer of pioneer neurons that overlies the ventricular zone. Recent studies have also suggested that Reelin controls neuronal orientation and polarized dendritic growth during this period of early cortical development. To explicitly characterize and quantify how Reelin controls this critical aspect of neurite initiation and growth we used a new *ex utero* explant model of early cortical development to selectively label a subset of L6 cortical neurons for complete 3-D reconstruction.

**Results:**

The total neurite arbor sizes of neurons in Reelin-deficient (*reeler* mutant) and Dab1-deficient (Reelin-non-responsive *scrambler* mutant) cortices were quantified and unexpectedly were not different than control arbor lengths (p = 0.51). For each mutant, however, arbor organization was markedly different: mutant neurons manifested more primary processes (neurites emitted directly from the soma) than wild type, and these neurites were longer and displayed less branching. *Reeler* and *scrambler* mutant neurites extended tangentially rather than radially, and the Golgi apparatus that normally invests the apical neurite was compact in both *reeler* and *scrambler* mutants. Mutant cortices also exhibited a neurite “exclusion zone” which was relatively devoid of L6 neuron neurites and extended at least 15 μm beneath the pial surface, an area corresponding to the marginal zone (MZ) in the wild type explants. The presence of an exclusion zone was also indicated in the orientation of mutant primary neurite and neuronal somata, which failed to adopt angles within ~20˚ of the radial line to the pial surface. Injection of recombinant Reelin to *reeler,* but not *scrambler*, mutant cortices fully rescued soma orientation, Golgi organization, and dendritic projection defects within four hrs.

**Conclusions:**

These findings indicate Reelin promotes directional dendritic growth into the MZ, an otherwise exclusionary zone for L6 neurites.

## Background

Identifying and characterizing the mechanisms responsible for dendritic growth and patterning are likely to provide insight into the etiology of some forms of epilepsy and mental retardation. For neurons developing in dissociated culture conditions, neurites adopt a default fate of ‘dendrite’ subsequent to axon specification [1]. In such neurons, dendrites extend mostly from the side of the soma opposite to that of the axon, but the neurons themselves do not collectively elaborate dendrites in any preferred direction. In contrast, neurons developing *in vivo* must not only specify axonal and dendritic identities, but also project their growing dendrites into the appropriate spatial regions for later synaptogenesis with presynaptic partners.

The importance of Reelin-Dab1 signaling in the process of cortical and hippocampal neuron dendritic development has been documented [2-7]. Reelin is a large glycoprotein secreted by Cajal-Retzius (CR) neurons in the preplate (PP) and marginal zone (MZ, future cortical layer 1) during early cortical development [8,9]. Reelin binding to its receptor molecules on migrating and differentiating cortical neurons leads to the tyrosine phosphorylation of the adaptor protein Disabled-1 (Dab1) [10-13]. Phospho-Dab1 then initializes a biochemical cascade involving both actin and microtubule dynamics [14,15] that controls cortical neuron migration and positioning. Recent studies have suggested an additional function of Reelin-Dab1 signaling in the control of neuronal orientation and dendritic outgrowth [4,5]. These processes involve Reelin-Dab1 suppression of an Lkb1/Stradα/Stk25 signaling complex that promotes axonal growth [16] in combination with a Rap1-dependent enhancement of N-cadherin at the plasma membrane of the developing dendrite [17-19]. ReelinDab1 signaling thus coordinates several disparate cellular events required for normal development of cortical projection neurons.

Although a contribution of Reelin-Dab1 signaling to cortical neuron dendritogenesis has been reported, the nature of this regulation are incompletely characterized. Depending on experimental paradigm, Reelin-Dab1 signaling potentially coordinates neurite growth support and/or chemotropic signaling required to orient dendritic elaboration by cortical neurons. To better define the role of Reelin-Dab1 signaling in the dendritic growth and spatial orientation of cortical neurons, we utilized a new, whole hemisphere explant model to examine dendritogenesis of layer 6 (L6) neurons. L6 neurons are the first neurons known to respond to Reelin during corticogenesis, and they do so while the cortical plate (CP) is forming [5,20]. We therefore subjected the nascent, growing dendritic arbors of L6 neurons to quantitative analyses. The results, which include analyses of L6 neurons in wild-type, *reeler* (Reelin-deficient), and *scrambler* (Dab1-deficient) brain explants, suggest a new model of Reelin-Dab1 signaling where Reelin may function as a factor that allows dendritic growth into the MZ.

## Results

### Embryonic day 13 electroporation targets prospective L6 neurons

**Figure 1:**
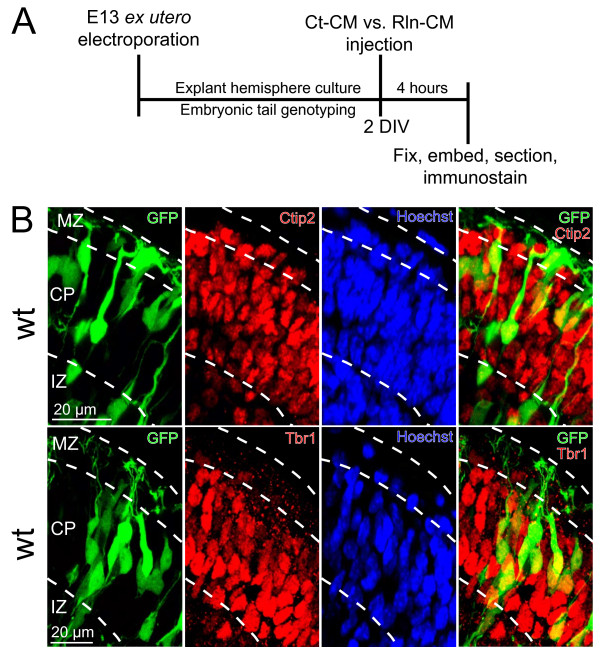
** Schematic of explant culture model.** (**A**) Brains from E13 embryos were electroporated *ex utero* with a GFP expression construct, dissected, and whole hemisphere explants were cultured on collagen-coated filters for 2 DIV. The embryos were genotyped and explants identified as wild type (wt), *reeler* (r/r,) or *scrambler* (s/s). After 48 hrs in culture, explants were injected with control conditioned media (Ct-CM) or Reelin conditioned media (Rln-CM), and cultured for an additional 4 hrs before fixation. (**B**) E13 *ex utero* electroporation targets prospective L6 neurons. Sections derived from wt explants were immunostained for the layer 5/6 transcription factors Ctip2 (top row) or Tbr1 (bottom row). The majority of the GFP + cells in the CP expressed Ctip2 (93%) and Tbr1 (93%). Scale bars: 20 μm in (B). For Ctip2, *n* = 363 GFP + positive neurons were totaled from 4 explants across 2 separate litters. For Tbr1, *n* = 326 GFP + neurons were totaled from 5 explants across 3 separate litters. All GFP + neurons included in these analyses were localized within 50 μm of the pial surface. Abbreviations: MZ, marginal zone; CP, cortical plate; IZ, intermediate zone.

Reelin-signaling has been shown to promote neuronal orientation and dendritogenesis of L6 neurons during preplate splitting [5], a period when L6 neurons also form a recognizable cortical layer between the marginal zone (MZ) and subplate (SP). However, this prior study was limited by the use of *Eomes::eGFP* transgenic mice [21] in which the GFP labeling density precluded detailed analysis of L6 neuron morphology. We therefore developed an *ex utero* electroporation (EUEP) approach to selectively label developing L6 cortical neurons. This method for labeling prospective L6 neurons allowed more consistent targeting of the electroporated region of the developing brain at this early time point than is typically possible with *in utero* electroporation approaches. The consistently targeted EUEPs permitted detailed resolution of the neurons and their neurites in the same area of developing mutant and wild type dorsomedial cortex (Figure 1A).

To confirm appropriate differentiation of L6 neurons targeted by electroporation, wild type explants electroporated on E13 were sectioned and immunostained for layer 5/6 markers Ctip2 and Tbr1 after 2 DIV. Within a defined area in the dorsomedial cortex (Field 1) that included cells no deeper than 50 μm below the pial surface, 93% GFP + neurons expressed the transcription factors Tbr1 (304/326) or Ctip2 (338/363) (Figure 1B). This expression of Tbr1 and Ctip2 in the superficially localized GFP + cells is consistent with a prior birthdating study [22] that reported L6 neurons in dorsomedial cortex undergo final mitosis on E13, the day on which we perform *ex utero* electroporation. Because electrofection of individual precursors is only successful if the cDNA is introduced within 6 hrs prior to M-phase [23], this birthdating analysis also indicates that GFP + neurons analyzed in Field 1 of the CP are developing L6 neurons. Thus the expression of layer-specific markers Tbr1 and Ctip2 indicate normal maturation of L6 neurons in these experimental conditions, with the timing of electroporation further supporting the L6 identity assignment.

### Disorganized cytoarchitecture of *reeler* cortical plate is rescued by reelin injection

**Figure 2:**
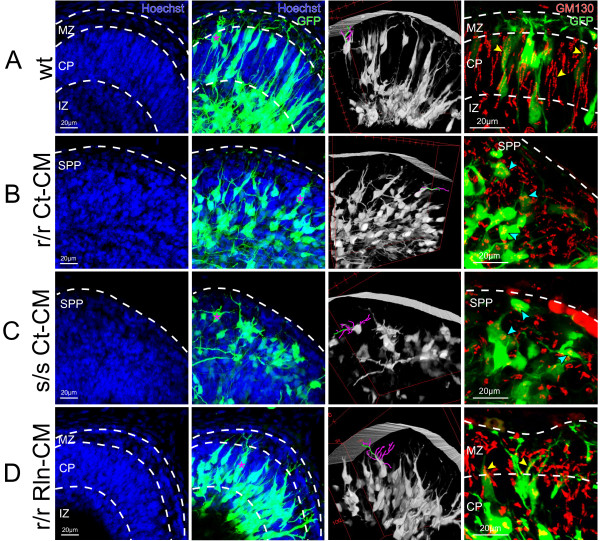
** Disorganized L6 neuronal morphology in*reeler*and*scrambler*explants and rescue by Reelin injection.** Hoechst nuclear stain (first column) reveals normal CP that forms during the 2 DIV culture period in wt (**A**) explants but not in r/r (**B**) or s/s (**C**) explants that were injected with Ct-CM. GFP expressing neurons (second column) show normal radially oriented neurites in wt (**A**) explants, but tangentially oriented processes in mutant (B, C) explants. 3-D rendering of imaged neurons (third column) confirms the radial and tangential orientations of L6 neurons observed in wt (**A**) and mutant (**B, C**) explants, respectively. Golgi/GM130 immunofluorescent labeling (fourth column) revealed elongated, pia-oriented Golgi colocalized to the apical neurite of L6 neurons in wt explants (yellow arrowheads; A), while condensed juxtanuclear Golgi were observed in both r/r Ct-CM and s/s Ct-CM mutant explants (blue arrowheads; **B** and **C**). (**D**) Within four hrs of exogenous Rln-CM injection into r/r explants, GFP + L6 neurons were oriented towards the pial surface in a clearly definable CP, demonstrated neurite elaboration into the MZ, and revealed elongated, pia-oriented Golgi colocalized to the apical neurite (yellow arrowheads). Purple asterisk represents traced neurons in 3-D rendering (third column). The single green line of the 3-D rendered traced neuron represents the primary neurite, while purple lines represent remaining neurites and corresponding branches. Scale bars: 20 μm in (**A-D**). Abbreviations: MZ, marginal zone; CP, cortical plate; SPP, superplate; IZ, intermediate zone.

To more accurately quantify Reelin’s role in neuronal orientation and dendritogenesis, we electroporated *reeler* (r/r), *scrambler* (s/s) and wild type (wt) control embryos on E13 with CAG-GFP plasmid and analyzed L6 neurons in Field 1 at 2 DIV (Figure 2). The *scrambler* mouse lacks the cytoplasmic adapter protein Disabled-1 (Dab1), a necessary component of the Reelin-receptor complex, and is a phenocopy of the *reeler* mouse [24-26]. In these experiments, Reelin-conditioned media (Rln-CM) or control-conditioned media (Ct-CM) were microinjected under the pia of the explant to determine the acute response of neurons to Reelin. Anti-Reelin immunostaining using the CR50 antibody [27] confirmed successful Reelin injection (Additional file 1).

In wild type explants, L6 neurons were radially organized towards the pial surface and displayed dendritic elaboration within the MZ (Figure 2A). This pial-directed orientation was confirmed after 3-D rendering of imaged volumes of the tissue (Additional file 2). In contrast, neurons in both *reeler* and *scrambler* mutant explants injected with Ct-CM appeared disorganized and were often not radially oriented. Although neurons in these mutants possessed extensive neurites, those neurites tended to extend tangentially rather than radially (Figure 2B, 2C). This was particularly apparent in the 3-D rendered images, which revealed that occasional radially oriented processes in the *reeler* and *scrambler* sections were often extending in the z-direction, and therefore tangentially oriented. (Additional files 3,4). Immunolabeling for the *cis*-Golgi marker GM130 [28] showed elongation of Golgi apparatus and Golgi outposts in wild type neurons (Figure 2A), but compacted and juxtanuclear Golgi in both the *reeler* and *scrambler* cortices (Figure 2B, 2C), as has been previously described [5,16]. Injection of recombinant Rln-CM into *reeler* explants rescued radial organization of the L6 neurons, allowed for Golgi elongation towards the pial surface and colocalization to the apical neurite, and promoted compaction of the CP (Figure 2D; Additional file 5). These cytoarchitectural features of *reeler* “rescued” explants were comparable to those observed in wild type explants, and are consistent with our prior findings [5]. In contrast, no rescue was observed after Rln-CM injection into *scrambler* explants (Additional file 6B, Additional file 7), consistent with the known Reelin-insensitivity of Dab1-deficient neurons. As a final control, Rln-CM injected into wild type explants revealed no differences in either cytoarchitecture or Golgi orientation/elongation, as compared to non-injected wild type controls (Additional file 6A, Additional file 8).

**Figure 3:**
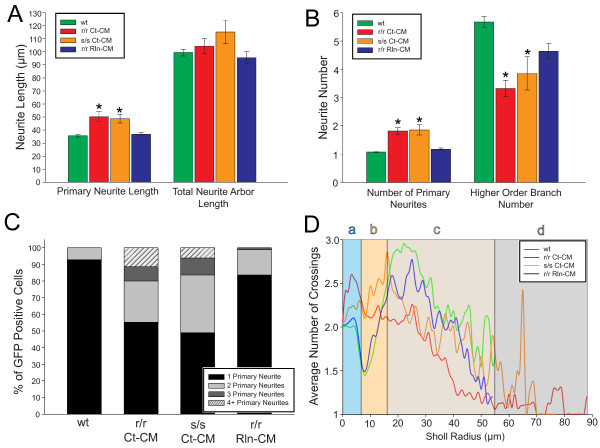
** Reelin-Dab1 signaling is required for normal neurite organization and branching but does not control total neurite length.** (**A**) L6 neurons in r/r Ct-CM and s/s Ct-CM explants had the same total neurite arbor length as wt neurons, despite longer primary neurites observed in mutant neurons. (**B**) Mutant neurons also displayed higher numbers of primary neurites (emitted from cell soma), but a decrease in higher order branch number as compared to wt controls. Rln-CM injection into r/r mutant explants rescued primary neurite length and branching patterns. (**C**) Categorization of neurons by primary neurite number and treatment condition. (**D**) 3-D Sholl analysis of traced GFP + neurons revealed consistent profiles of neurite crosses vs. radius for r/r Rln-CM neurons and wt neurons at all radial domains (a-d). Error bars denote standard error of the mean. Kruskal-Wallis one-way ANOVA on ranks with post-hoc Dunn tests were performed between treatment conditions. * p < 0.05, as compared to wt controls. wt analysis: 209 neurons reconstructed from 22 explants across 9 litters. r/r Ct-CM analysis: 105 neurons reconstructed from 7 explants across 5 separate. s/s Ct-CM analysis: 49 neurons reconstructed from 3 explants across 3 litters. r/r Rln-CM analysis: 86 neurons reconstructed from 8 explants across 4 separate litters.

To quantify the response of L6 neurons to Reelin we performed complete 3-D reconstructions of neurons using Simple Neurite Tracer [29]. Reelin-Dab1 signaling has a well-established role in promoting dendritic growth of hippocampal neurons [6] and is known to promote dendritic growth of upper layer neurons into the MZ [7]. We first quantified total neurite arbor length and surprisingly found no difference between neurons in *reeler* and *scrambler* mutant explants injected with Ct-CM vs. wild type (104.4 and 115.2 μm vs. 99.4 μm, p = 0.51; Figure 3A). The organization of the neuronal arbor, however, was significantly different in mutant explants. Mutant L6 neurons possessed significantly longer primary neurites (i.e. neurites extending directly from the cell body) as compared to wild type (50.3 and 48.8 μm vs. 35.6 μm, p < 0.05; Figure 3A) and exhibited more primary neurites per neuron (1.8 and 1.9 vs. 1.1, p < 0.05; Figure 3B). Consistent with these data, approximately 90% of all wild type control neurons had a single primary neurite, with the remaining 10% displaying no more than two primary neurites (Figure 3C). In comparison, about 50% of neurons in mutant explants possessed a single primary neurite, with the remaining 50% having two, three, or greater than four primary neurites (Figure 3C). This increase in primary neurites was accompanied by a significant decrease in higher-order branch number in the mutants as compared to wild type controls (3.3 and 3.9 vs. 5.7, p < 0.05) (Figures 3B). Injection of Rln-CM into *reeler* mutants achieved a near-complete rescue of the aberrant neurite morphology: both primary neurite length and number of primary neurites were reduced to wild type values (36.7 μm vs. 35.6 μm, p > 0.05, and 1.2 vs. 1.1, p > 0.05, respectively; Figures 3A,B). Similarly, the percentage of GFP + neurons with two, three, or greater than four primary neurites were observed to sharply decrease, with 83% of all analyzed neurons having only a single primary neurite, comparable to wild type explants (Figure 3C). These observations were confirmed by 3-D Sholl analysis (Figure 3D), which revealed that Reelin-injection recovered a near-wild type profile of branching within 4 hrs. The quantitative analyses of the mutant dendritic arbors thus produced two surprises: the mutant neurons had a normal amount of neurite, but possessed more primary neurites and a simplified neurite arbor. All of these differences could be rapidly rescued by Reelin injection.

### Reelin injection rescues somal and primary neurite orientation

**Figure 4:**
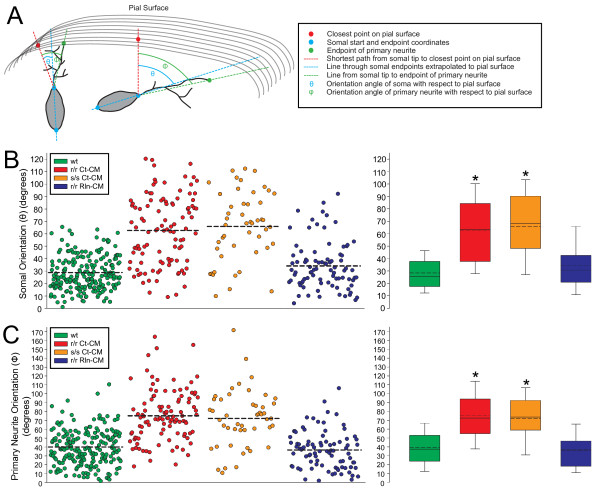
** Reelin-Dab1 signaling is required for orientation of neuronal somata and primary neurites.** (**A**) The angles of the neuronal soma (θ) and primary neurite (φ) with respect to the shortest line from the somal tip to the pial surface (red line in A) were measured in 3-D using a custom MATLAB script. Only GFP + neurons within 50 μm of the pial surface were included for analysis. Scatter and box plots of somal orientation (**B**) and primary neurite orientation (**C**) in all four explant conditions. Both r/r Ct-CM and s/s Ct-CM neurons demonstrated significant somal and primary neurite misorientation compared to wt neurons. Injection of Rln-CM significantly rescued both somal and primary neurite orientation angles in r/r mutant explants. Dashed lines on both scatter and box plots represent mean values; solid lines on box blots represent median values. Box plots display upper and lower quartiles; whiskers represent 90th and 10th percentiles. Kruskal-Wallis one-way ANOVA on ranks with post-hoc Dunn tests were performed between treatment conditions. * p < 0.05, as compared to wt controls. wt analysis: 209 neurons from 22 explants across 9 litters. r/r Ct-CM analysis: 105 neurons from 7 explants across 5 separate litters. s/s Ct-CM analysis: 49 neurons from 3 explants across 3 litters. r/r RlnCM analysis: 86 neurons from 8 explants across 4 separate litters.

To quantify the orientation of neuronal somata and neurites, a skeletonized version of the L6 neuron along with isocontour lines representing the pial surface of the explant, were imported into a custom MATLAB script. This script determined the shortest path extending from the neuronal soma to the closest point of the rendered pial surface. The angles made by the primary neurite (φ) and the long axis of the neuronal soma (θ) with respect to that shortest line were then determined (Figure 4A; See Methods). These angles potentially represent distinct biological processes (i.e., neurite navigation and cellular orientation/polarity) and therefore were both measured. The average somal orientation in wild type explants was 28.4 ± 1.1°, whereas *reeler* and *scrambler* mutant explants displayed significantly misoriented somal orientation angles of 62.6 ± 2.7° and 65.9 ± 4.0°, respectively (p < 0.05; Figure 4B). Similarly, the primary neurite angles of mutant neurons were significantly misoriented (75.1 ± 2.8° and 72.0 ± 4.6°, respectively) as compared to wild type controls (39.4 ± 1.5°, p < 0.05; Figure 4C). Interestingly, the correlation coefficient (R) between φ and θ ranged from 0.55 (wild type) to 0.45 (*reeler* mutant) suggesting that the orientation angles of the primary neurite and soma are not independent and may share biological mechanisms (Additional file 9). Within four hrs of Reelin injection however, both somal and primary neurite orientation of L6 neurons in *reeler* mutant explants recovered to control values (34.2 ± 2.1° and 36.5 ± 2.3°, respectively, p > 0.05; Figure 4B, 4C).

### Reelin mutations decrease neurite elaboration in the developing marginal zone

Both *reeler* and *scrambler* neurons largely avoided orientation angles within ~20° of the pial surface, whereas a large percentage of neurons in wild type and rescued explants demonstrate angles below 20° (Figure 4B, 4C). If Reelin functions as a chemotropic signal, the absence of Reelin signaling might be expected to produce random orientation of the neurite and soma, including angles below 20 degrees. The non-random distribution of angles suggests that in the absence of Reelin, the subpial region may be relatively impermissive for neurite growth. We therefore investigated if the mutant subpial region (to a depth of approximately 15 μm) had significantly diminished L6 neurites as compared to control.

**Figure 5:**
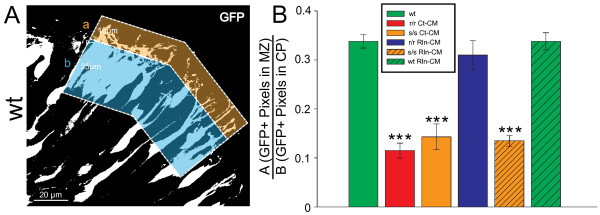
** L6 neurons in mutant explants have decreased neurite elaboration into the MZ.** (**A**) Representation of threshold analysis for the measurement of neurite elaboration into the MZ. Threshold was determined for the flattened, 8-bit grayscale image, and the ratio of GFP + pixels in the MZ (*a*; ~ 15 x 100 μm ROI) to GFP + pixels in the CP (*b*; ~ 35 x 100 μm ROI) was determined for each treatment condition. (**B**) Both r/r Ct-CM and s/s Ct-CM L6 neurons revealed decreased GFP + signal (i.e. neurite elaboration) into the MZ as compared to wt controls and r/r Rln-CM explants. Ratios were compared across images acquired from 18 wt explants, 8 r/r Ct-CM explants, 4 s/s Ct-CM explants, and 8 r/r Rln-CM explants. Error bars denote standard error of the mean. One-way ANOVA with post-hoc Bonferroni t-tests were performed between treatment conditions. *** p < 0.001, as compared to wt controls.

To determine the amount of neurite present in the MZ, images from all experimental conditions were first thresholded (see Methods). The number of suprathreshold pixels in a region of interest (ROI) extending 15 μm immediately below the pial surface and extending 100 μm parallel to the pia (15 × 100 μm ROI) was quantified and normalized to the suprathreshold pixels in the subadjacent CP (35 × 100 μm ROI) (Figure 5A). Wild type explants had two- to three-fold greater neurite elaboration (i.e. suprathreshold GFP + pixels) in the subpial region as compared to both *reeler* and s*crambler* mutant explants, as well as *scrambler* explants injected with Rln-CM (p < 0.001; Figure 5B). Within four hrs of Reelin injection, levels of GFP + pixels in *reeler* explants recovered completely (p = 1.0). Lastly, GFP + pixels in the subpial region of wild type explants injected with Rln-CM were not significantly different than control wild type sections (p = 1.0). This analysis suggests that L6 neurites require Reelin-Dab1 signaling to penetrate an apparent “exclusion zone” extending ~15 μm below the pial surface.

**Figure 6:**
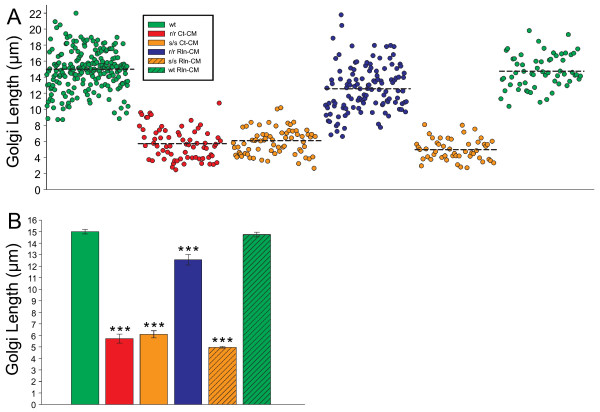
** Reelin-Dab1 signaling is required for Golgi extension.** Sections from explants were immunolabeled with the *cis-*Golgi marker GM130 and imaged. (**A**) Scatter plots of proximal-to-distal Golgi lengths measured in each experimental condition. L6 neuron Golgi lengths were compact in both r/r Ct-CM and s/s Ct-CM explants as compared to elongated wt and partially rescued (i.e. elongated) Golgi in r/r Rln-CM explants. Rln-CM injection into s/s or wt explants(s/s Rln-CM and wt Rln-CM) had no effect on Golgi length in comparison to Ct-CM injections. (**B**) Bar graph representation of measured Golgi lengths**.** Dashed lines in scatter plots represent mean values; error bars denote standard error of the mean. Golgi lengths were compared across images acquired from 18 wt explants, 7 r/r Ct-CM explants, 5 s/s Ct-CM explants, 9 r/r Rln-CM explants, 5 s/s Rln-CM explants, and 6 wt Rln-CM explants. One-way ANOVA with post-hoc Bonferroni t-tests were performed between treatment conditions. *** p < 0.001, as compared to wt controls.

To determine whether the decrease in neurite growth in the MZ observed in mutant explants was accompanied by a corresponding alteration in Golgi morphology (Figure 2; Additional file 6), we quantified the proximal-to-distal length of the Golgi in sections immunolabled with the *cis*-Golgi marker GM130 [28]. Wild type Golgi lengths averaged 15.0 ± 0.8 μm, whereas *reeler* and *scrambler* mutant Golgi were significantly compacted, having lengths of 5.7 ± 1.0 μm and 6.1 ± 0.7 μm (p < 0.001), respectively (Figure 6A, 6B). Rln-CM injection into the *reeler* mutant explant partially rescued Golgi length to 12.6 ± 1.4 μm, while Rln-CM injection into *scrambler* or wild type explants had no effect on Golgi length in comparison to Ct-CM injections (p = 1.0). Collectively, these analyses indicate that Reelin-Dab1 signaling may not have any direct function in the initial phase of dendritogenesis *per se*, as the total neurite arbor size is the same between mutants and wild type explants. An alternative model is therefore suggested in which Reelin-Dab1 signaling is required for Golgi deployment and directed neurite growth into the MZ.

## Discussion

In this study we have quantitatively examined a fundamental aspect of Reelin signaling, namely its role in stimulating dendritic growth. Contrary to expectations, immature L6 neurons do not require Reelin-Dab1 signaling to initiate or support the earliest phase of dendritic growth during preplate splitting, as the total neurite arbor size was the same in mutant and wild type cortex explants. Instead, our data suggest that Reelin-Dab1 signaling has a major function in promoting pial-directed dendritic growth into the marginal zone.

Despite progress in understanding the biochemical cascade initiated by Reelin [30], how Reelin-signaling stimulates preplate splitting and dendritogenesis *in vivo* has been unclear. It is generally believed that Reelin positions neurons, possibly by controlling their migration or translocation [7,31,32]. However, prior studies showed that during early cortical development L6 neurons are equivalently positioned with respect to the pial basal lamina in *reeler* and wild type cortices [5,33]. These finding suggest that the initial positioning of L6 neurons is independent of Reelin-signaling, and the ectopic positioning of L6 neurons observed in mature mutant cortex evolves during later corticogenesis. This observation was exploited in the present study, in which we successfully electroporated prospective L6 neurons in mutant and wild type cortices and quantitatively examined their morphology two days later, at equivalent positions in the developing CP (Additional file 10).

Complete dendritic reconstruction of L6 neurons in *reeler* and *scrambler* explants revealed equivalent total neurite arbor size as compared to controls (~110 mm). The mutant neurons, however, possessed more primary neurites and less branching than wild type controls (Figures 2,3). This finding is consistent with two features of our prior study of Dab1-suppressed L2/3 neurons [7]: Dab1-suppressed neurons displayed simplified dendritic arbors, and the apical neurites were less elaborate in the MZ. However, in this prior study we were unable to completely reconstruct the dendritic arbor of individual cells due to the high density of electroporation labeled cells and intermingling of their large arbors. Similarly, in a prior study of L6 neuron development we were unable to determine total arbor size due to the high density of eGFP labeling in the transgenic (*Eomes::eGFP*)gsat mouse [5].

Examination of cultured hippocampal neurons has shown that Reelin-Dab1 signaling can enhance dendritic growth and branching [6]. However, there are some important differences with the current report. First, the quantitative component of the earlier work involved long term culture of hippocampal, rather than cortical neurons. Second, the overall neurite length was not different at 2 DIV, the time period of our analysis, but differences emerged by 4 DIV and were maximal by 6 DIV. The earlier study thus points to a long term, but slower Reelin-dependent mechanism that does promotes dendritic growth in hippocampal neurons.

The most dramatic differences between wild type and Reelin-Dab1 deficient L6 neurons in this study involved the mutant neurons’ (1) extra primary neurites and (2) misorientation of the long axis of the neuronal soma and the primary neurite. The cell body and primary processes of neurons in the *reeler* and *scrambler* mutant explants were tangentially organized compared to the radially organized wild type neurons (Figure 4). Other studies have reported that Reelin-Dab1 deficiency can lead to disorientation of neurons: L6 neurons showed misoriented somata and Golgi during the period of preplate splitting [5]; hippocampal neurons in CA fields 1-3 are disoriented [6]; and Purkinje cells have misoriented neurites during early cerebellar development [4]. A specific function of Reelin-Dab1 signaling as a spatial organizer of neuronal somata and dendritic arbors is therefore indicated for early cortical development.

The importance of the Golgi apparatus in both cellular polarity and neurite formation has been previously highlighted [34]. For developing pyramidal neurons, the Golgi first localizes to the site of axon initiation [35,36] and later is re-positioned to the *opposite* side of the neuron, at the site of apical neurite initiation [37-39]. Golgi outposts are then distributed into the apical neurite and can promote dendritic growth and branching [37,39,40].

The molecular events that drive Golgi positioning depend on neuronal polarity signaling, principally the pro-axogenic signaling pathway Lkb1/Stradα that is antagonized by the pro-dendritogenic Reelin-Dab1 pathway [16]. The protein kinase Lkb1, the sterile kinase related protein Stk25, and the pseudokinase Stradα cooperatively promote neuronal polarization and axon specification [16,41-43]. Stk25 regulates Golgi morphology through the Golgi matrix protein GM130 [44], probably by regulating GM130-mediated fusion of ER-to-Golgi vesicles [45,46]. Since Golgi position anticipates the location of process extension [37-39,47], a central role of Reelin-Dab1 signaling may be to regulate Golgi deployment that in turn supports neurite extension. This antagonistic regulation by Lkb1/Stradα/Stk25 and Reelin-Dab1 may effectively commit the neuron to the temporal deployment of axons, and then dendrites, during the combined processes of neuronal migration and neurite elaboration [16].

If Reelin-Dab1 signaling overcomes an inhibitory signal localized to the MZ, this might occur by stabilization of neurites invading the MZ. In this model the inhibitory signal *de-stabilizes* only those neurites that enter the MZ, and Reelin-Dab1 signaling would counteract this destabilization. While we propose that the inhibitor is localized to the MZ, Reelin-Dab1 signaling could occur either in the MZ by Reelin binding to receptors on the nascent neurites or on the soma itself where Reelin receptors can also be found [48]. Reelin-Dab1 neurite stabilization might result from altered n-cofilin phosphorylation [15] in conjunction with Rap1-dependent N-cadherin surface expression and increased adhesion of the nascent neurite in the MZ [18,19]. This increase in adhesion might in turn enhance microtubule deployment and Golgi investment in the new neurite serving to enhance both somal translocation and dendritic growth towards the pia [5,16].

In the absence of Reelin-Dab1 signaling, the neurites of L6 neurons that initially invade the MZ, experience inhibition and may not adhere. This postulated lack of adhesion prevents the establishment of both a primary neurite and later, pia-oriented elongation of the soma. In the *reeler* cortex the nucleus and soma of the L6 neuron elongates in the direction of the tangentially extending primary neurite and thus the soma becomes misoriented. The consequence of Reelin-Dab1 deficiency may be slightly different for later born neurons that migrate on radial glial fibers and are therefore already pia-oriented by virtue of their attachment to the radial glial process. For these migrating neurons, the absence of Reelin-Dab1 signaling also prevents the leading process from adhering to the MZ, but in this case the leading process adhesion may be a necessary prerequisite for glial-independent somal translocation [7,18], the steady movement of the nucleus into the primary process [32]. Somal orientation (elongation of the nucleus in the direction of the primary neurite) and translocation (movement of the nucleus into the primary neurite) could be mechanistically related in that both could involve dynein-based forces pulling on the nucleus [49-51]. Thus Reelin-Dab1 deficiency for both L6 neurons and later born neurons prevents the establishment and elaboration of a primary neurite in the MZ. However, the consequence of this common deficit could be different for the two neuronal populations: misorientation of L6 neurons and failed somal translocation of upper layer neurons.

The preceding model does not completely explain Reelin’s role in preplate splitting as additional mechanisms may be involved in the formation of a coherent layer 6 between the MZ and SP. We speculate that simultaneous with nascent process formation, the adhesive properties of the neuronal soma may change with increased surface expression of N-cadherin, leading to altered cell-cell adhesion and the cell sorting activities that may underlie preplate splitting [5]. Absent Reelin-Dab1 signaling, L6 neurons do not orient or coalesce appropriately, leading to knock-on consequences in cell positioning. Importantly, the initial function of Reelin-Dab1 signaling in this model is to facilitate the directed growth of the dendrite into the MZ.

## Conclusions

**Figure 7:**
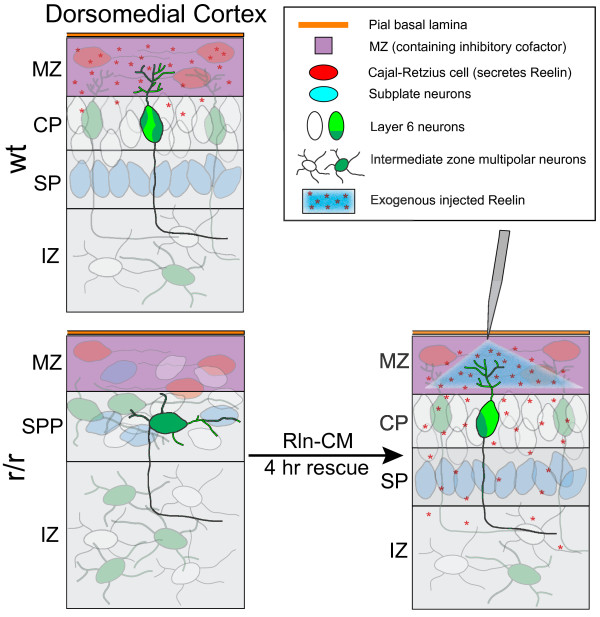
** Reelin-Dab1 signaling in L6 neuron orientation and dendritogenesis.** In the absence of Reelin signaling (r/r explant, lower panel), L6 neurons had the same total neurite arbor length as wild type (wt explant, upper panel). However, neurons in the mutant had more primary neurites that were longer and less branched than control primary neurites. Additionally, the primary neurites and neuronal somata in the mutant were oriented tangentially rather than radially. Notably, L6 neuron neurites failed to penetrate the MZ in the mutant despite their normal length. These observed defects are hypothesized to be consistent with the operation of an inhibitory signal (purple zone) present in the MZ that is counteracted by Reelin signaling.

Our detailed analysis of orientation angle and GFP + neurite content in the MZ suggest a new, yet primary function of Reelin-Dab1 signaling during early cortical development: the negation of a neurite inhibiting factor localized to the MZ (Figure 7). If Reelin-Dab1 signaling functioned to stimulate directed neurite outgrowth, then the *absence* of Reelin-Dab1 signaling would be expected to result in primary L6 neurites with random orientations. Instead, the distribution of angles in both *reeler* and *scrambler* mutants is non-random, with a pronounced deficiency of processes oriented to within 20˚ of the pial surface. This observation, in conjunction with a ~60% decrease in GFP + neurite content within an “exclusion zone” extending 15 μm immediately below the pia of mutant explants, suggests the presence of an exclusion zone which may be impermissive for dendrite growth in the absence of Reelin-Dab1 signaling. While the molecular basis for this exclusion zone is unknown, its existence is parsimonious with the results of our quantitative analyses. We speculate that a normal function of such an exclusion zone might be to prevent inappropriate connections between axonal projections coursing through layer 1 and a subset of cortical neurons defined by their inability to respond to Reelin.

## Methods

### Mice

All animal protocols were reviewed and approved by the IACUC of SUNY Upstate Medical University. *reeler* (B6C3Fe *a/a-Reln*^*rl*/+^, Jackson Laboratories, Bar Harbor, ME) and *scrambler* (Jackson Laboratories, Bar Harbor, ME) heterozygote mice were mated to produce the *reeler* (r/r) and Dab1-deficient *scrambler* (s/s) mutant embryos used for the described experiments. Data from wild type and heterozygote embryos from both *reeler* and *scrambler* litters were compiled into a single data set, designated as wild type (wt). Embryonic day 0 (E0) was designated as the day of plug discovery.

### Explant cultures

Embryonic day 13 (E13) embryos were electroporated *ex utero* with 0.33 mg/ml of a pCAG-GFP construct [52]. The electroporations consisted of 5 pulses of 30 V, each pulse lasting 50 msec and separated by a 950 msec interval. The anode was positioned near the midline of the head and the cathode was positioned under the chin. Embryos and explants were immersed in ice cold 1X Hank’s Balanced Salt Solution during electroporation, dissection, and culturing. Whole brains were removed and hemisected with meninges intact, and left hemispheres were cultured medial side down on 3 μm pore size collagen-coated polytetrafluoroethylene filters (Transwell-COL, Corning) using a previously described protocol [5]. Briefly, the explants were cultured in DMEM-F12 medium plus GlutaMAX and supplemented with 1% G5, 2% B27, and 1X Penicillin and were then maintained in a high oxygen environment (95% O2/5% CO2) at 37 °C for the duration of the experimental procedure [12,53]. All cell culture reagents were from Invitrogen (Carlsbad, CA).

### Production of conditioned medias

A HEK293 cell line was used to produce control conditioned media (Ct-CM) and a stable HEK293 cell line [54] was used to produce Reelin-conditioned media (Rln-CM). Conditioned medias from near-confluent cultures were collected after 48 hrs of incubation in serum-free Opti-MEM media supplemented with 1X GlutaMAX and 1X Pen/Strep. Amicon Ultra 100,000 molecular weight cut off filters (Millipore, Billerica, MA), were used to concentrate the conditioned medias, which were then brought to a 10-fold concentration in fresh explant culture media (DMEM-F12 plus aforementioned supplements) as described [5]. Conditioned media were usually collected and used on the same day for injections.

### Reelin injection/bath application

Wiretrol pipettes (Drummond Scientific Co., Broomall PA) were pulled to a fine point, the tip broken with jeweler’s forceps, and placed in a holder attached to a micromanipulator (Narishige, Japan). On E15, controlled injections of either Rln-CM or Ct-CM (~0.5 - 1 μl) were made into multiple medial and lateral points in each explant using a pneumatic Picopump (PV820, World Precision Instruments, Inc., Sarasota, FL). Post-injection, half of the culture media was exchanged with fresh Rln-CM or Ct-CM, and ~200 μl of Rln-CM or Ct-CM was added on top of the explants. Injection and bath application periods were brief (< 20 minutes) and carried out at room temperature under a dissection microscope (Olympus SZX12). The explants were then returned to the high oxygen environment and cultured for an additional 4 hrs.

### Histology/histochemistry

Post-injection E15 explants were drop fixed for 1 hr in 4% paraformaldehyde/Pagano solution (250 mM Sucrose, 25 mM MgCl_2_, 2.5 mM KCl, HEPES 7.4). The tissue was embedded in 10% calf gelatin (Aldrich), post fixed for 24 hrs in 4% paraformaldehyde/Pagano solution and sectioned at 100 μm using a vibratome. Floating sections were sequentially incubated overnight with primary and secondary antibodies diluted in PBS (with 0.5% Triton-X-100 and 2% BSA). Anti-CR50 (1:500, MBL), anti-Tbr1 (1:1000, Abcam), anti-GM130 (1:100, BD Transduction Laboratories) and anti-Ctip2 (1:500, Abcam) were used. The appropriate Alexa Fluor 555-conjugated IgG (1:500, Molecular Probes) and Alexa Fluor 647-conjugated IgG (1:500, Molecular Probes) secondary antibodies were used. Hoechst 33342 (2 μg/ml, Molecular Probes) was used to counterstain nuclei. Images were acquired using a Zeiss LSM510 laser scanning 2-photon confocal microscope (SUNY Upstate Medical University Center for Bioresearch Imaging).

### Imaging and analysis

Z-series were collected with a Zeiss LSM510 laser scanning confocal microscope (SUNY Upstate Medical University Center for Bioresearch Imaging). Images were collected at 1 μm z-step intervals. The images were imported into FIJI (www.fiji.sc), an open source distribution of Image J (Wayne Rasband, NIH). Individual cells were traced and 3-D reconstructions made using the open source Simple Neurite Tracer plugin in the FIJI package [29]. Only neurons within 50 μm of the pial surface were traced and included for MATLAB analysis. Total neurite arbor size and branching was measured within FIJI. A custom script was run on MATLAB (Version 7.14, Mathworks Inc. Natick, MA) to determine the primary neurite and cell soma orientation with respect to the shortest path tangent to the pial surface. First, the pial surface was manually traced in each z-step of the stack. These contour lines were then imported into MATLAB and interpolated as a surface. The skeletonized neuron from Simple Neurite Tracer was imported and the shortest distance between the rendered neuronal soma and the interpolated surface was determined. The angles between the shortest line to the surface and either the primary neurite (φ) or the long axis of the neuronal soma (θ) were then determined in 3-D. Sholl analysis was run on each traced neuron, using the center of the cell body as the origin. The Sholl analysis was executed within FIJI using the linear method with a sphere separation of 1 μm, and no normalization.

### GFP fluorescent intensity analysis

In order to quantify the relative amount of GFP + neurites present in the MZ, the first 10 z-steps of previously acquired 1 μm interval z-series were imported into NIH ImageJ (W. Rasband, National Institutes of Health, Bethesda, MD) and flattened into a single optical section. The images were then thresholded to a value of 80 (~30% over baseline, full scale 255). For each image, the number of above-threshold pixels was measured in defined regions of interest (ROI) corresponding to the MZ in wild type sections (15 × 100 μm box; containing neurites) and subadjacent CP (35 × 100 μm box; containing cell bodies). The ratio of the number of GFP + pixels in the MZ to the number of GFP + pixels in the CP was calculated for each image, and compared between all experimental conditions.

### Golgi length quantification

To quantify the length of GM130 immunolabeled Golgi apparatus, the first 15 z-steps (1 μm interval z-series) were imported into ImageJ and flattened into a single section using a maximum brightness projection. In order to avoid quantification of Cajal-Retiuz cell Golgi in the MZ, a 35 × 100 μm ROI was overlayed on the flattened image, with the top of the box beginning approximately 15 μm below the pial surface. Using the line draw tool, Golgi were traced from the proximal to the distal point. A minimum of 5 Golgi were quantified per explant, and compared between all experimental conditions.

### Statistical analyses

For all orientation and neurite morphology measurements, Kruskal-Wallis One Way Analysis of Variance (ANOVA) on Ranks with Dunn’s method for pairwise multiple comparison procedures were run to determine significant differences between any experimental. These tests were run after ascertaining a failure of data normality by the ShapiroWilk test. GFP pixel intensity ratios and Golgi lengths between all experimental conditions passed the Shapiro-Wilk normality test, so a One Way ANOVA with post-hoc Bonferroni t-tests were run on these data.

## Abbreviations

MZ, Marginal zone; CR, Cajal-Retzius; CP, Cortical plate; SP, Subplate; IZ, Intermediate zone; L6, Layer 6; L2/3, Layer 2/3; Rln, Reelin; Ct, Control; CM, Conditioned media; wt, Wild type; DIV, Day in vitro; GFP, Green fluorescent protein; ROI, Region of interest.

## Competing interests

The authors declare that they have no competing interests.

## Authors’ contributions

ECO and RSO designed the project. RSO performed the experiments. DAC, WRZ, RRW and SML participated in the development of the analytic approach. CJMU wrote the MATLAB script with input from ECO. RSO, DAC and ECO wrote the manuscript. All authors read and approved the final manuscript.

## Supplementary Material

Additional file 1 **(pdf Localization of Reelin in E15 cultured explants.** Using CR50 immunofluorescent staining (second column), CR50 immunosignal was strongest in the MZ with minor diffusion into the CP in wt (A), s/s Ct-CM (C), wt Rln-CM (E), and s/s Rln-CM (F) explants. Despite the presence of Reelin in s/s Ct-CM (C) and s/s Rln-CM (F) explants, GFP + L6 neurons remained misoriented relative to the pial surface due to mutations in the Dab1 cytoplasmic adapter protein and subsequent inability to respond to Reelin protein. As expected, r/r Ct-CM explants (B) revealed an absence of Reelin protein, while r/r Rln-CM “rescued” explants (D) demonstrated the presence of injected Reelin. Scale bars: 20 μm in (A-F). Abbreviations: MZ, marginal zone; CP, cortical plate; SPP, superplate; IZ, intermediate zone. Click here for file

Additional file 2 **(mov 3-D rendering of GFP + L6 neurons and overlying pial surface in a wt explant.** Wt neuronal somata and primary neurites were radially oriented towards the pial surface. The traced cell is a representative GFP + L6 neuron. The single green line of the 3-D rendered traced neuron represents the primary neurite, while purple lines represent remaining neurites and corresponding branches. Click here for file

Additional file 3 **(mov 3-D rendering of GFP + L6 neurons and overlying pial surface in a r/r explant injected with Ct-CM.** Mutant neuronal somata and primary neurites were tangentially oriented and parallel to the pial surface. The traced cell is a representative GFP + L6 neuron. The single green line of the 3-D rendered traced neuron represents the primary neurite, while purple lines represent remaining neurites and corresponding branches. Click here for file

Additional file 4 **(mov 3-D rendering of GFP + L6 neurons and overlying pial surface in a s/s explant injected with Ct-CM.** Mutant neuronal somata and primary neurites were tangentially oriented and parallel to the pial surface. The traced cell is a representative GFP + L6 neuron. The single green line of the 3-D rendered traced neuron represents the primary neurite, while purple lines represent remaining neurites and corresponding branches. Click here for file

Additional file 5 **(mov 3-D rendering of GFP + L6 neurons and overlying pial surface in a r/r explant injected with Rln-CM.** Injection of Rln-CM into r/r explants promoted both somal and primary neurite orientation towards the pial surface (similar to wt controls), as well as neurite arbor elaboration into the MZ. The traced cell is a representative GFP + L6 neuron. The single green line of the 3-D rendered traced neuron represents the primary neurite, while purple lines represent remaining neurites and corresponding branches. Click here for file

Additional file 6 **(pdf Cytoarchitecture of cortical plate and L6 neuronal morphology in Reelin-injected control explants.** Similar to wt explants, wt explants injected with Rln-CM (A) demonstrate a clearly definable cortical plate by Hoechst nuclear counterstain (first column), while s/s mutant explants injected with Rln-CM (B) display cortical plate abnormalities similar to those observed in both r/r Ct-CM and s/s Ct-CM L6 neurons, consistent with the non-rescuable *scrambler* phenotype. GFP expressing L6 neurons (second column) shown normal radially oriented neurites with elaboration into the MZ in wt Rln-CM (A) explants, but tangentially oriented processes in s/s Rln-CM (B) explants. 3-D rendering of imaged neurons (third column) confirms the radial and tangential orientations of L6 neurons observed in wt Rln-CM (A) and s/s Rln-CM (B) explants, respectively. Golgi/GM130 immunofluorescent labeling (fourth column) revealed elongated, pia-oriented Golgi with deployment down the apical dendrite of GFP + neurons in wt Rln-CM explants (yellow arrowheads; A), while condensed, juxtanuclear Golgi were observed in s/s Rln-CM explants (blue arrowheads; B). Purple asterisks represent traced neurons in 3D rendered snapshots (third column). Scale bars: 20 μm in (A-B). Abbreviations: MZ, marginal zone; CP, cortical plate; SPP, superplate; IZ, intermediate zone. Click here for file

Additional file 7 **(mov 3-D rendering of GFP + L6 neurons and overlying pial surface in a s/s explant injected with Rln-CM.** Injection of Rln-CM into s/s explants had no effect on somal or primary neurite orientation. Neuronal soma and primary neurites remained tangentially oriented, similar L6 neurons in s/s explants injected with Ct-CM. The traced cell is a representative GFP + L6 neuron. The single green line of the 3-D rendered traced neuron represents the primary neurite, while purple lines represent remaining neurites and corresponding branches. Click here for file

Additional file 8 **(mov 3-D rendering of GFP + L6 neurons and overlying pial surface in a wt explant injected with Rln-CM.** Injection of Rln-CM into wt explants had no effect on somal or primary neurite orientation. Neuronal soma and primary neurites remained radially oriented, similar L6 neurons in uninjected wt explants. The traced cell is a representative GFP + L6 neuron. The single green line of the 3-D rendered traced neuron represents the primary neurite, while purple lines represent remaining neurites and corresponding branches. Click here for file

Additional file 9 **(pdf Comparison of somal orientation (θ) and primary neurite orientation (φ) for all traced L6 neurons.** Linear regressions of somal orientation (θ) vs. primary neurite orientation (φ) revealed moderate correlations, ranging from 0.55 (wt explants) to 0.45 (r/r Ct-CM explants), suggesting that primary neurite and somal orientation angles may not represent distinct biological processes, but rather may share biological mechanisms. Click here for file

Additional file 10 **(pdf Average distance from the pial surface between explant conditions.** (A) The average depth from the pial surface of quantified GFP + L6 neurons from wt, r/r Ct-CM, and s/s Ct-CM explants were found to lie within one cell body length. All neurons included for analysis were within 50 μm of the pial surface (lower limit of counting box represented by dashed line). Average depth of MZ quantified over all explant conditions was 15 μm. Green oval represents schematic of average cell body length (15 μm) quantified across all explant conditions. Dashed lines of box plots denote mean values; solid lines denote median values. Box plots display upper and lower quartiles; whiskers represent 90th and 10th percentiles. Abbreviations: MZ, marginal zone; CP, cortical plate. Click here for file
