# Supplementary figures and images for: Layer 6 cortical neurons require Reelin-Dab1 signaling for cellular orientation, Golgi deployment, and directed neurite growth into the marginal zone
Source: Neural Dev. 2012 Jul 7;7:25. doi: 10.1186/1749-8104-7-25 (PMC3466444; doi:10.1186/1749-8104-7-25)

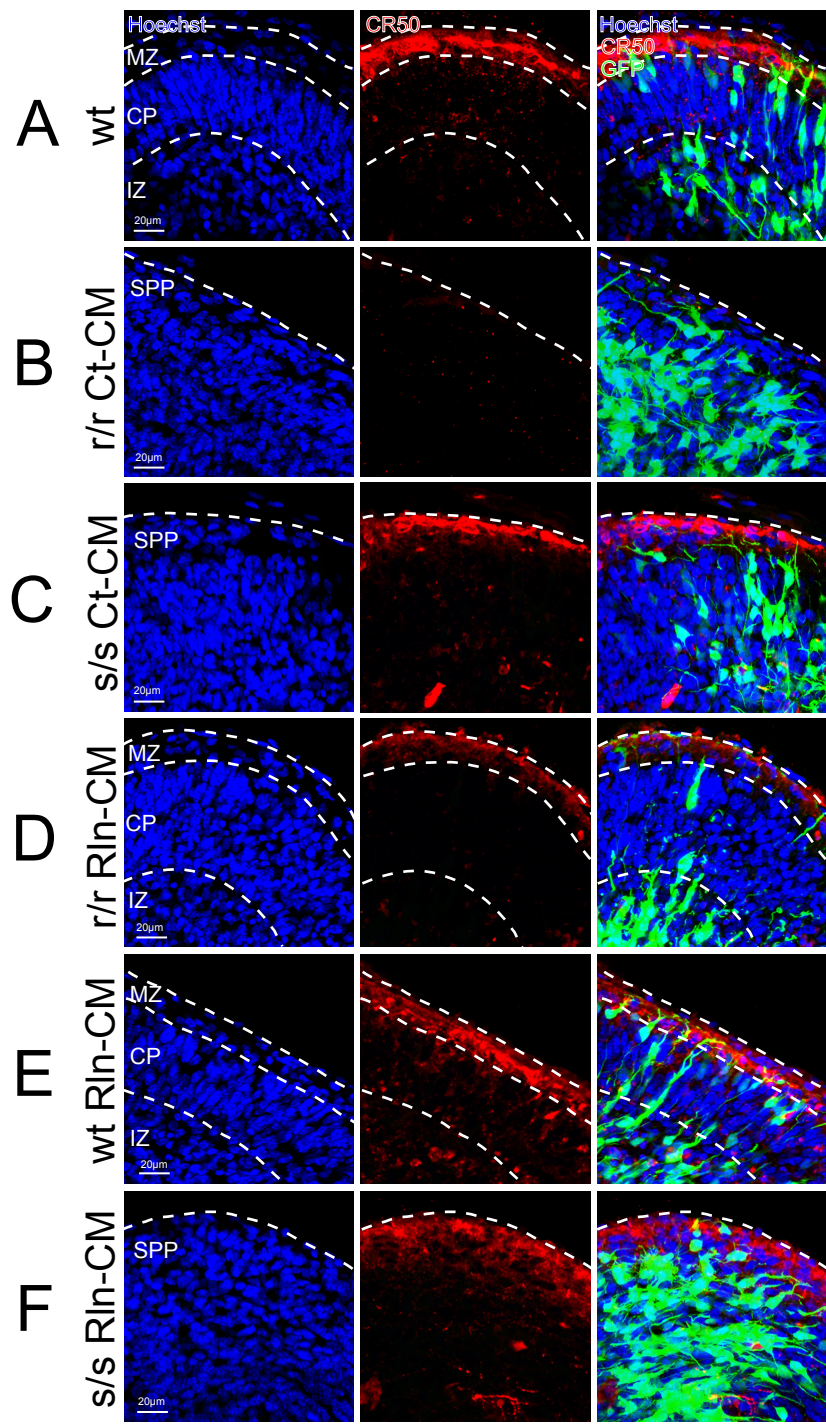

Supplement: Additional file 1 — (pdf Localization of Reelin in E15 cultured explants. Using CR50 immunofluorescent staining (second column), CR50 immunosignal was strongest in the MZ with minor diffusion into the CP in wt (A), s/s Ct-CM (C), wt Rln-CM (E), and s/s Rln-CM (F) explants. Despite the presence of Reelin in s/s Ct-CM (C) and s/s Rln-CM (F) explants, GFP + L6 neurons remained misoriented relative to the pial surface due to mutations in the Dab1 cytoplasmic adapter protein and subsequent inability to respond to Reelin protein. As expected, r/r Ct-CM explants (B) revealed an absence of Reelin protein, while r/r Rln-CM “rescued” explants (D) demonstrated the presence of injected Reelin. Scale bars: 20 μm in (A-F). Abbreviations: MZ, marginal zone; CP, cortical plate; SPP, superplate; IZ, intermediate zone. [file 1749-8104-7-25-S1.pdf]

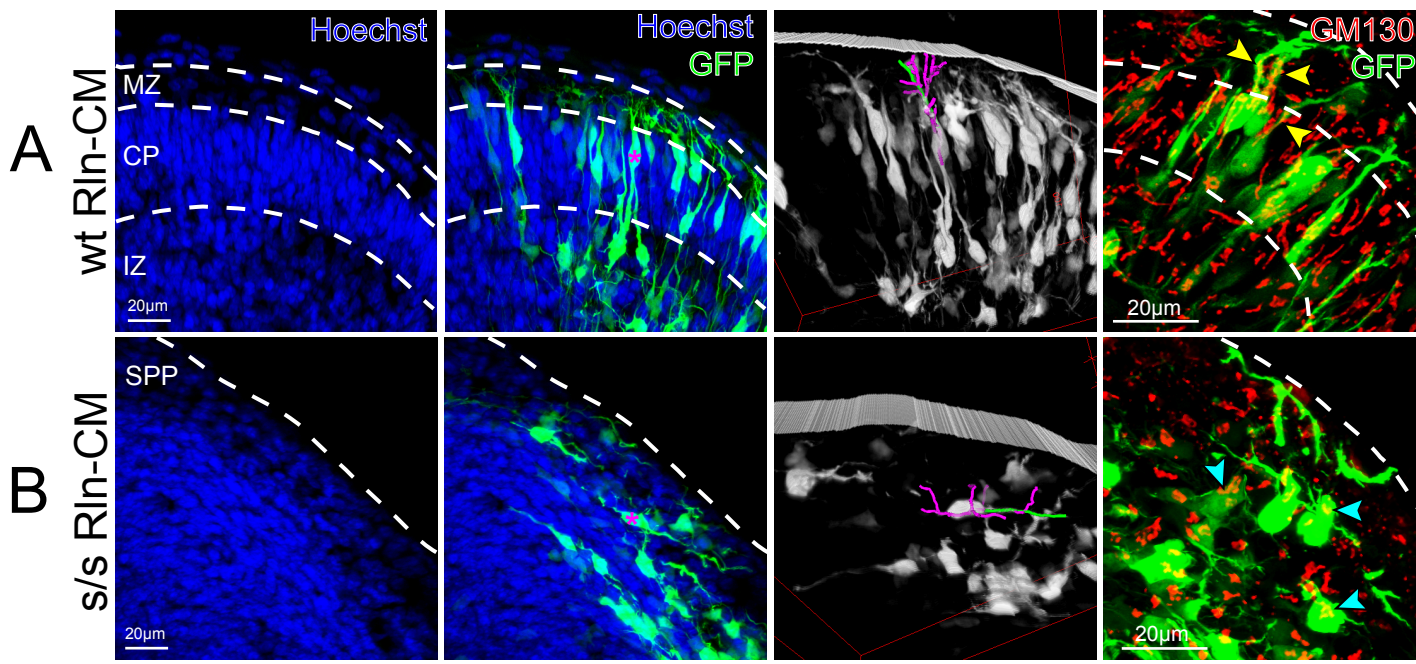

Supplement: Additional file 6 — (pdf Cytoarchitecture of cortical plate and L6 neuronal morphology in Reelin-injected control explants. Similar to wt explants, wt explants injected with Rln-CM (A) demonstrate a clearly definable cortical plate by Hoechst nuclear counterstain (first column), while s/s mutant explants injected with Rln-CM (B) display cortical plate abnormalities similar to those observed in both r/r Ct-CM and s/s Ct-CM L6 neurons, consistent with the non-rescuable scrambler phenotype. GFP expressing L6 neurons (second column) shown normal radially oriented neurites with elaboration into the MZ in wt Rln-CM (A) explants, but tangentially oriented processes in s/s Rln-CM (B) explants. 3-D rendering of imaged neurons (third column) confirms the radial and tangential orientations of L6 neurons observed in wt Rln-CM (A) and s/s Rln-CM (B) explants, respectively. Golgi/GM130 immunofluorescent labeling (fourth column) revealed elongated, pia-oriented Golgi with deployment down the apical dendrite of GFP + neurons in wt Rln-CM explants (yellow arrowheads; A), while condensed, juxtanuclear Golgi were observed in s/s Rln-CM explants (blue arrowheads; B). Purple asterisks represent traced neurons in 3D rendered snapshots (third column). Scale bars: 20 μm in (A-B). Abbreviations: MZ, marginal zone; CP, cortical plate; SPP, superplate; IZ, intermediate zone. [file 1749-8104-7-25-S6.pdf]

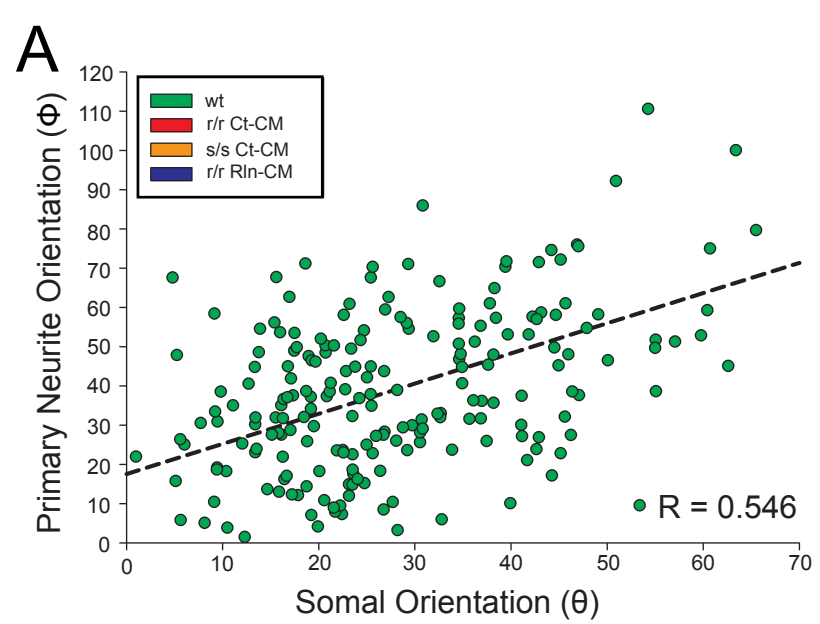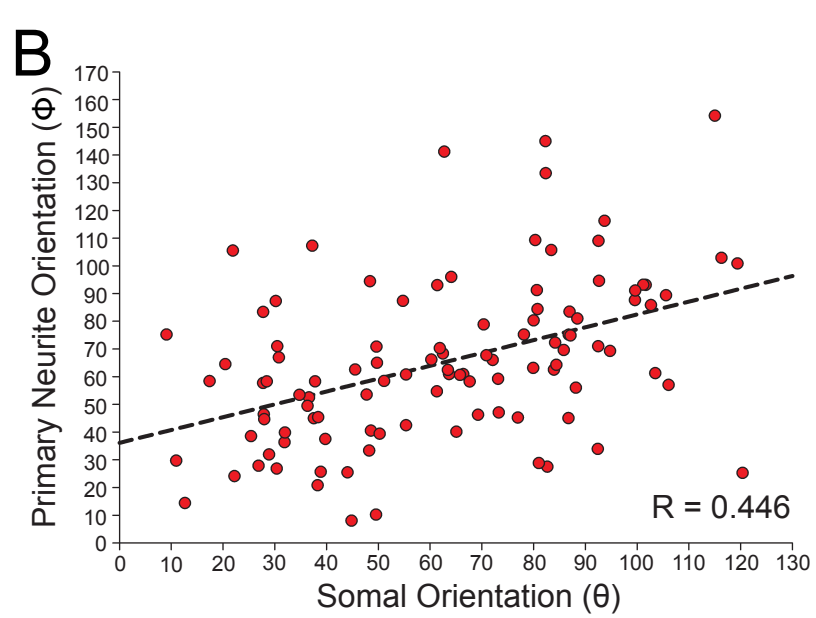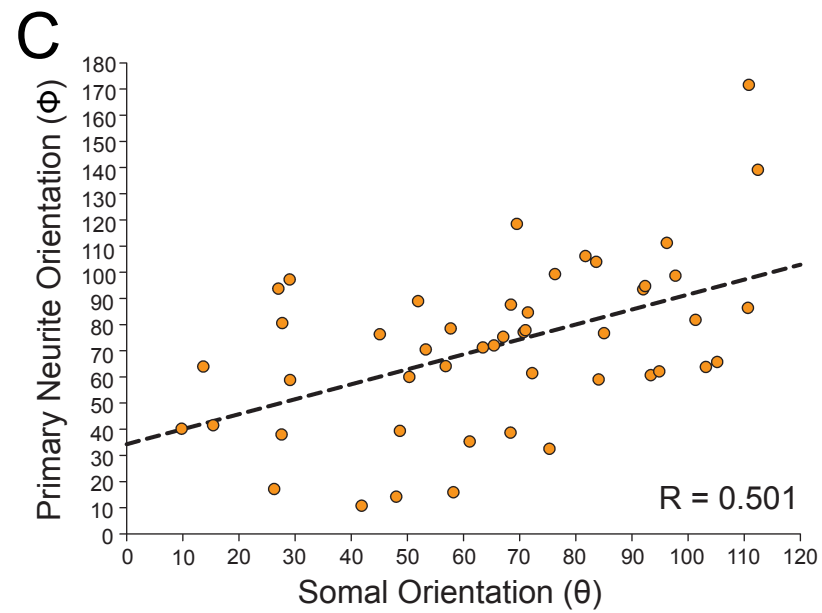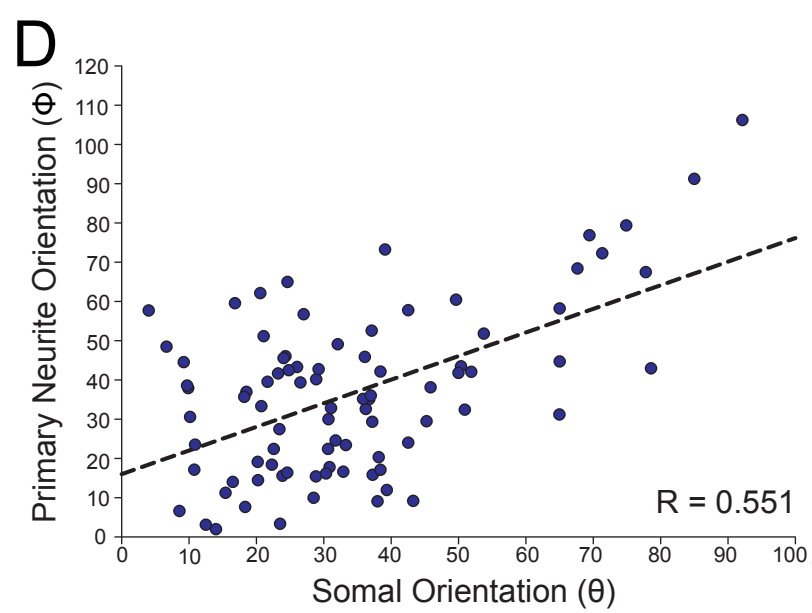

Supplement: Additional file 9 — (pdf Comparison of somal orientation (θ) and primary neurite orientation (φ) for all traced L6 neurons. Linear regressions of somal orientation (θ) vs. primary neurite orientation (φ) revealed moderate correlations, ranging from 0.55 (wt explants) to 0.45 (r/r Ct-CM explants), suggesting that primary neurite and somal orientation angles may not represent distinct biological processes, but rather may share biological mechanisms. [file 1749-8104-7-25-S9.pdf]

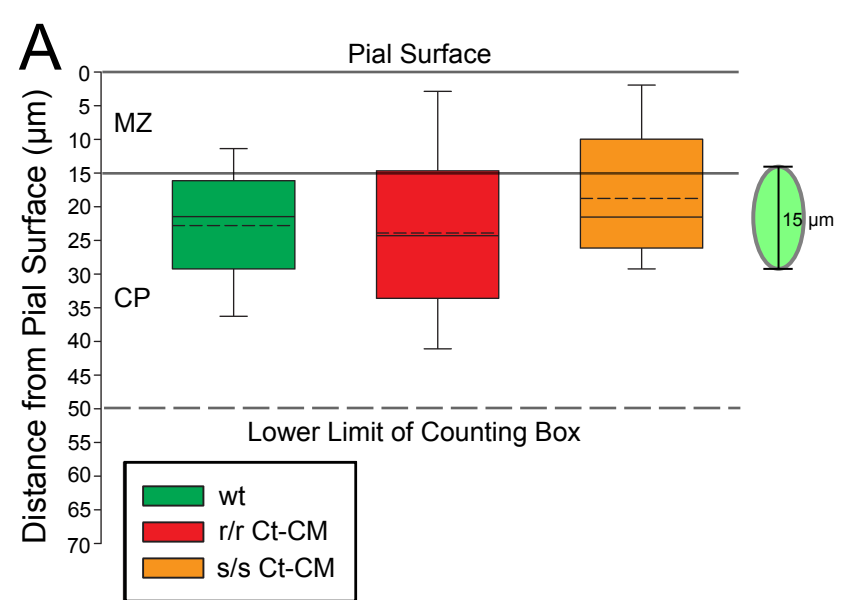

Supplement: Additional file 10 — (pdf Average distance from the pial surface between explant conditions. (A) The average depth from the pial surface of quantified GFP + L6 neurons from wt, r/r Ct-CM, and s/s Ct-CM explants were found to lie within one cell body length. All neurons included for analysis were within 50 μm of the pial surface (lower limit of counting box represented by dashed line). Average depth of MZ quantified over all explant conditions was 15 μm. Green oval represents schematic of average cell body length (15 μm) quantified across all explant conditions. Dashed lines of box plots denote mean values; solid lines denote median values. Box plots display upper and lower quartiles; whiskers represent 90th and 10th percentiles. Abbreviations: MZ, marginal zone; CP, cortical plate. [file 1749-8104-7-25-S10.pdf]
